# The Association between Carotid Intima-Media Thickness and the Duration of Type 1 Diabetes in Children

**Published:** 2014-06

**Authors:** Masoud Pezeshki Rad, Donya Farrokh, Rahim Vakili, Mozhgan Omidbakhsh, Mohaddeseh Mohammadi

**Affiliations:** 1 Vascular and Endovascular Surgery Research Center, Department of Radiology; 2 Department of Radiology; 3 Department of Pediatrics, Imam Reza Hospital, Faculty of Medicine, Mashhad University of Medical Sciences, Mashhad, Iran

**Keywords:** Type 1 Diabetes Mellitus; Carotid Intima-Media Thickness; Vascular Diseases; Atherosclerosis

## Abstract

***Objective:*** Carotid intima-media thickness (cIMT) has been known as a criterion of generalized atherosclerosis and a marker of cardiovascular disease progression in many studies which can be measured by ultrasound using high-resolution device.

***Methods:*** This is a case-control study. A total of 40 children (16 males and 24 females) with type1 diabetes mellitus and control group consisting of equal numbers (17 males and 23 females) who were otherwise healthy were included in the study from May 2007 to January 2008. The two groups were age matched, with the mean age of 10.56±3.21 years in control group and 10.67±4.18 years in diabetic patients. Left and right cIMT were measured by ultrasound.

***Findings*** ***:*** There was a significant difference between case and control subjects in terms of mean cIMT (*P*<0.001). cIMT was significantly higher in the diabetic group. Among variables including age, BMI and diabetes, diabetes was the only influential parameter in this respect. The mean time length of type 1 diabetes in our diabetic group was reported 4.24±3.02 years, with a minimum of four months and a maximum of ten years. There was a statistically significant difference between the two diabetic patients with below and above four years of disease duration (*P*=0.03 for right carotid artery and *P*=0.01 for left carotid artery).

***Conclusion:*** cIMT has been identified as an early indicator of atherosclerosis in many studies. It increases in patients with type 1 diabetes as the disease progresses and this can be followed by macro and microvascular atherosclerotic changes.

## Introduction

Diabetes mellitus is the most prevalent metabolic disease of childhood and adolescence^[^^1^^,^^2^^]^, and type 1, caused by autoimmune destruction of the pancreas is the most common type in this age group^[^^3^^]^. Individuals with diabetes have two-fold to four-fold increased risk of developing atherosclerotic disease. Type 1 diabetes is an important risk factor for cardiovascular disease. Children with diabetes mellitus are considered as high-risk patients and need special attention^[^^4^^,^^5^^]^. Type 1 diabetes deserves more importance as it tends to affect younger age groups, and as a result, for longer period of time^[^^6^^,^^7^^]^. 

 Carotid intima-media thickness (cIMT) has been known as a criterion of generalized atherosclerosis and a marker of cardiovascular disease progression in many studies^[^^8^^]^. Apart from invasive imaging techniques for the assessment of patients with type 1 diabetes such as coronary angiography, non-invasive procedures including echocardiography and ultrasonography, have been proven safer, most cost-effective and generally acceptable especially for younger age groups.

 High-resolution ultrasound is a reliable method for detecting early structural and functional atherosclerotic changes in the arterial wall. Although diabetic patients have more risk to have peripheral atherosclerosis in comparison with control subjects, the association between cIMT and the duration of the underlying condition, type 1 diabetes , still remains to be established^[^^9^^,^^10^^]^. 

 In this study, we aimed to compare the results of cIMT measurement by B-mode ultrasonography in type 1 diabetic children with a non-diabetic control group to determine whether there is any association between the duration of type 1 diabetes and cIMT.

## Subjects and Methods

This is a case-control study. Pediatric patients with type1 diabetes aged 10.56±3.21 (range 6-15) years participated in this study. The control group consisted of healthy children matched for age, sex, weight and BMI. 40 children (16 males and 24 females) with type 1 diabetes mellitus who presented to the endocrinology clinic in our institution, and equal number of children in the control group who were referred to the same center for a variety of other insignificant complaints were evaluated over one-year from May 2007 to January 2008.

 Patients with known cardiovascular problems, hyperlipidemia, metabolic conditions other than diabetes and not consent were excluded from our study. The subjects in control group were void of any history of diabetes or cardiovascular disease. 

 This study was approved by the ethics committee of our institution. The parents/ guardians of patients and control group signed an informed consent form.

 Demographic data, clinical as well as laboratory findings, including glucose level and HBA_1C_ recorded over the last year were elicited.

 A Doppler and real-time carotid artery ultrasound was performed for all patients and control group. Ultrasound studies were performed by a single experienced radiologist who was blinded to the patients’ clinical history. All ultrasound studies were performed with a high-resolution ultrasound device (Siemens G40) equipped with a 7 to 10 MHz linear array transducer. cIMT was measured at three distinct points in each right and left common carotid artery and the place of measurements was fixed in every ultrasound study. The cIMT was defined as the distance from the leading edge of the front echogenic line to the second hypoechoic line from the upper layer of the adventia.

 Data are expressed as mean±SD. All recorded data were analyzed in SPSS18, using t-test and non-parametric equity tests. A *P-*value <0.05 was considered statistically significant. A range of descriptive-analytical methods were used including central and distributive indicators, frequency distribution, analytical statistical methods, Chi^2^ to compare qualitative variables including age, diabetes, t-test to assess quantitative variables including duration of diabetes, age, glucose level, HbA_1C_ and BMI in both groups (if there was normal distribution) and equal non-parametric test (if there was not normal distribution). Analyses were carried out (*P*<0.05) and results presented.

***Findings***

A total of 40 children consisting of 16 (40%) males and 24 (60%) females with type 1 diabetes and 40 age matched normal controls consisting of 17 (42.5%) males and 23 (57.5%) females participated in the study (*P*=0.8). 

**Fig. 1:**
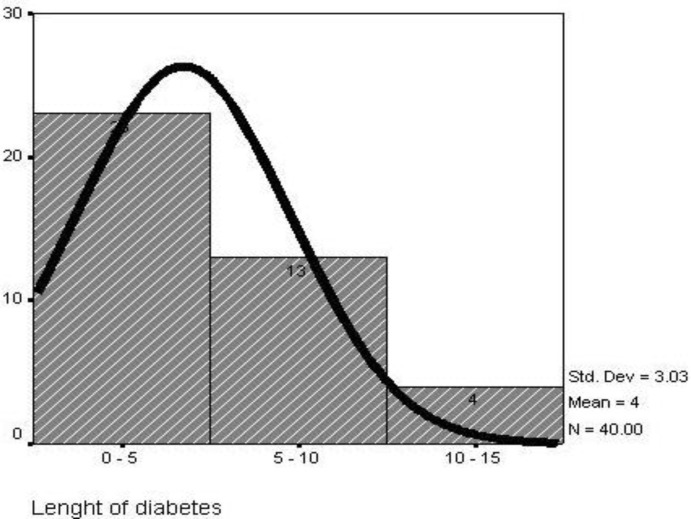
Length of diabetes in children with type1 diabetes

**Fig. 2:**
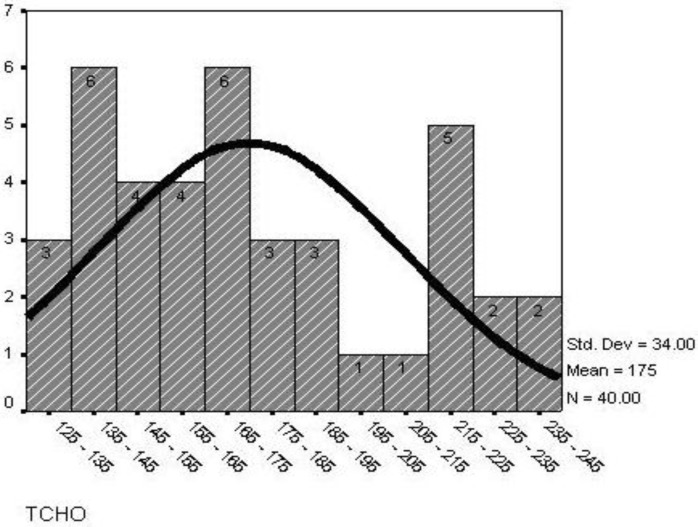
Distribution of glucose level in children with type 1 diabetes

**Fig. 3:**
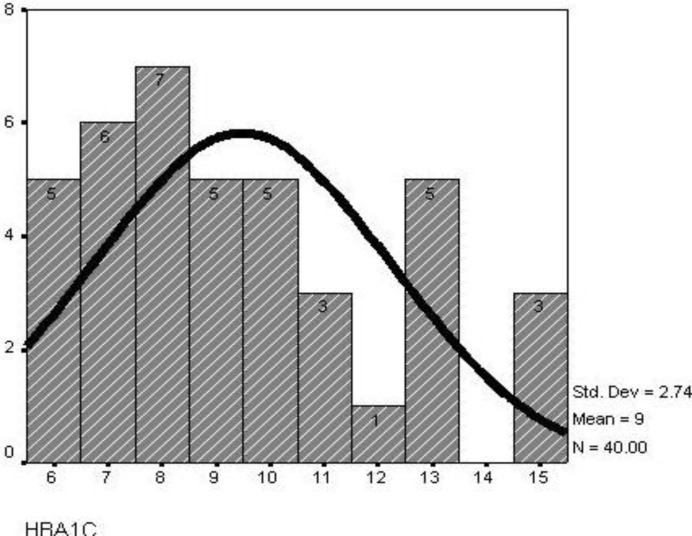
Distribution of HbA_1c_ in children with type1 diabetes

 Mean age of diabetic patients was 10.67±4.18 years, while that of the control subjects was 10.56±3.21 years. The mean BMI in diabetic patients was 17.67±2.84 while in the control subjects it was 15.34±3.81 (*P*=0.003). The mean duration of type 1 diabetes mellitus was 4.24±3.02 years (4 months to 10 years) (Fig. 1). Mean glucose level was 174.9±34.0 (125mg-240mg) (Fig. 2) and mean HBA_1c_ was 9.48±2.73 (5.5%-15.4%) (Fig. 3). 

 There was a significant difference in cIMT measurements between the patient group and control subjects, using independent t-test (*P*<0.001). The intima-media thickness was considerably higher in the diabetic patients than in the control group (Table 1).

 Among multiple variables including age, BMI and diabetes that were evaluated, diabetes was the only one that could affect the left and right cIMT at all spots (Table 2). Our patients' group were divided into two subgroups based on the duration of diabetes. In 23 children the duration of diabetes was below 4 years and in 17 patients it was above 4 years. The mean±SD values of cIMT of both carotid arteries were measured. Mean cIMT was consistent in both groups (Table 3). Both groups showed a significant difference in IMT in common carotid arteries (Mann-Whitney test, *P*=0.03 and 0.01 for right and left carotid artery respectively). 

 cIMT of both common carotid arteries showed significant difference in two subgroups (with duration of diabetes below and above 4 years) (*P*=0.03 for right carotid, *P*=0.01 for left carotid).

## Discussion

The primary landmark of atherosclerosis has been shown to be endothelial dysfunction as well as intima-media thickness (IMT) increase in almost all arteries^[^^11^^]^. Macrovascular disease leading to cardiovascular complications remains the endpoint of type 1 diabetes mellitus in childhood.

**Table 1:** Mean carotid intima-media thickness between the two groups

**Carotid Intima-Media Thickness**		**Control**	**Diabetic **	***P. value***
**Right Carotid**	First segment	0.44 (0.09)	0.49 (0.10)	<0.001
	Second segment	0.44 (0.10)	0.50 (0.09)	
	Third segment	0.42 (0.08)	0.48 (0.10)	
	Mean three segments	0.43 (0.06)	0.49 (0.07)	
**Left Carotid**	First segment	0.44 (0.09)	0.51 (0.10)	<0.001
	Second segment	0.45 (0.07)	0.50 (0.09)	
	Third segment	0.44 (0.08)	0.50 (0.09)	
	Mean three segments	0.44 (0.04)	0.50 (0.08)	

 Atherosclerotic lesions develop slowly but continuously leading to the main causes of mortality in diabetic patients. Anatomical studies revealed that atherosclerosis can start in childhood, particularly if inflicted with type 1 diabetes^[^^8^^]^. In diabetic children the presence of subclinical atherosclerotic disease as a precursor of macrovascular complications has been observed. Children with type 1 diabetes mellitus are prone to cardiovascular diseases, which are more likely to occur later in life given the advancements in therapeutic measures are disregarded.

 Within the present study, IMT was evaluated in both right and left common carotid artery. The mean right and left cIMT was 0.49 and 0.5 mm respectively, which is more than that reported by Ab-del-Ghaffar et al^[^^12^^]^ and less than that of Nathan et al^[^^13^^]^. Data reported here show that atherosclerotic burden (assessed by cIMT) is already increased in children with type 1 diabetes compared with an age, sex and BMI-matched control group.

 In our study cIMT was significantly higher in patients with diabetes type 1 compared with that of control group (*P*<0.001); similar results were shown by Gul^[^^9^^]^ in Turkey in 2010 (*P*<0.0001).

 In our study the male to female ratio was almost two to three in both patients and controls. Other similar studies did not show any significant sex preference. In Harrington's study 66 patients with type 1 diabetes were evaluated for cIMT measurement, of whom 37 cases were males^[^^10^^]^. Also in Dejaberi's study 83 of 150 children with type 1 diabetes were males^[^^3^^]^. In Distiller's study 76 of 148 children with type 1 diabetes were males^[^^14^^]^. 

 The mean age of our patients was 10.67±4.18 years whereas Harrington et al reported that the mean age of their patients was 14.1±2.5 years^[^^10^^]^. As it had been expected, the mean BMI in children with type 1 diabetes was higher than that of the control group in our study.

 Harrington et al reported a substantial rise in aortic intima-media thickness, however they failed to generalize this to the carotid artery possibly due to the fact that the former changes come prior to the latter in the course of diabetes^[^^10^^]^. 

 Gul et al performed a cohort study on diabetic patients and they failed to establish a correlation between cIMT and mean HBA_1c_, or between cIMT and the efficacy of blood sugar control by insulin in the first year. They attributed the accelerated rate of atherosclerosis to gradual accumulation of glycosylation products^[^^9^^,^^15^^]^. It takes at least a few years to produce atherosclerotic changes^[^^13^^]^. 

**Table 2:** The result of logistic regression model of the right and left carotid intima-media thickness based on the diabetes

**Variables**	**Coefficient**	**OR**	**CI 95%**	***P. *** **value**
**Diabetes**	-1.29	0.810	(0.11-0.70)	0.007
**Diabetes**	-2.51	0.231	(0.02-0.30)	<0.001

OR: Odds Ratio; CI: Confidence Interval

**Table 3:** Mean carotid intima-media thickness based on the duration of diabetes

**Carotid Intima-Media Thickness**	**<4 years**	**≥** **4 years **	***P. *** **value**
**Right Carotid**	0.48 (0.08)	0.49 (0.09)	0.03
**Left Carotid**	0.51 (0.07)	0.52 (0.05)	0.01

 The association between cIMT, diabetes duration and age was evaluated in our study and by other research groups. Aside from age as a variable that can influence cIMT, the duration of diabetes is a relevant factor for cIMT increase. This is also consistent with other similar cohort studies^[^^16^^,^^17^^]^, as strict blood sugar control by insulin improved cIMT after six years^[^^10^^]^. 

 Longer disease duration early in life might enhance the metabolic effects of diabetes on the vascular system and result in earlier onset and accelerated progression of atherosclerosis. However, with increasing age other classical cardiovascular risk factors may play an additional role in diabetic children.

 Longitudinal cIMT measurements revealed progression in subclinical atherosclerosis during a four year period in diabetic children and adolescents (0.58±0.75, *P*<0.001) as was shown by Dalla Pozza et al in Germany in 2011 (*P*<0.001)^[^^18^^]^. 

 Some limitations of our study have to be addressed. As the similar studies, relatively small sample size was considered as the limitation of our study which was due to small number of participants who met the inclusion criteria. So these results cannot be generalized to a majority of patients with type 1 diabetes. Also observational study cannot establish causality.

 We were able to show that cIMT is elevated in children with type 1 diabetes in comparison to a healthy control group. Other risk factors such as microalbuminuria or the time of the diagnosis need to be investigated in further studies^[^^12^^]^. A further limitation is the loss-to-follow-up of these patients in this study. Despite the above limitations mentioned above, the results of our study indicate that children with type 1 diabetes mellitus show increased cIMT values and cIMT can still be regarded as a primary marker of atherosclerotic changes, and that ultrasound is a non-invasive method for cIMT measurements in patients with type 1 diabetes.

## Conclusion

Adolescence has been identified as a critical time in determining risk of future vascular complications in type 1 diabetes mellitus. Interventions to prevent these cardiovascular complications will be most effective if implemented at a young age.

 cIMT has been regarded as an early indicator of atherosclerosis in type 1 diabetes. CIMT increases as the disease progresses, causing a range of macro and microvascular atherosclerosis-induced complications. Therefore ultrasound cIMT measurements can be considered as a non-invasive screening test and follow-up method in diabetic children. Finally we recommend annually measurements which can provide useful data and could be evaluated in longitudinal studies.

## Authors’ Contribution

Concept and design: M. Pezeshki Rad, D. Farrokh and R. Vakili 

Acquisition of the data: M. Omidbakhsh 

Data analysis and interpretation: M. Pezeshki Rad and D. Farrokh 

Manuscript preparation: M. Mohammadi

Critical revision of the manuscript: M. Pezeshki Rad, D. Farrokh, R. Vakili, M. Omidbakhsh, and M. Mohammadi

All authors approved final version of the manuscript.
